# Assessment of anxiety disorders in students starting work with coronavirus patients during a pandemic in Podlaskie Province, Poland

**DOI:** 10.3389/fpsyt.2022.980361

**Published:** 2022-08-11

**Authors:** Klaudia Paula Czorniej, Elzbieta Krajewska-Kułak, Wojciech Kułak

**Affiliations:** ^1^Students' Scientific Society, Department of Integrated Medical Care and Department of Pediatric Rehabilitation and Center of Early Support for Handicapped Children “Give a Chance,” Medical University of Białystok, Białystok, Poland; ^2^Department of Integrated Medical Care, Medical University of Białystok, Bialystok, Poland; ^3^Department of Pediatric Rehabilitation and Center of Early Support for Handicapped Children “Give a Chance,” Medical University of Białystok, Bialystok, Poland

**Keywords:** anxiety, social phobia, professional work, pandemic, students

## Abstract

**Background:**

Anxiety disorders are considered the sixth most important factor resulting in non-fatal health loss in the world. Moreover, they are among the first ten causes of years lived with disability (YLD) across the globe. Important clinical disorders include e.g., panic disorder, social anxiety disorder, generalized anxiety disorder and specific phobia.

**Objectives:**

The study aimed to analyse the occurrence of level anxiety in students who start work at the time of the COVID-19 pandemic, with relation to the socio-demographic factors and health status, vaccination, conovirus infection, assistance of a psychologist or psychiatrist in the past, and using tranquilizers.

**Methods:**

The study involved 255 students from Poland starting work with coronavirus patients during the pandemic. It was conducted using our own questionnaire, the Liebowitz Social Anxiety Scale (LSAS) and the State-Trait Anxiety Inventory (STAI).

**Results:**

Fifty-one percent of subjects demonstrated symptoms of mild to severe social phobia. Level of trait anxiety among students correlated significantly with age and gender (females). The level of social anxiety in the evaluated students was significantly correlated with marital status, the self-assessment of the experienced fear, self-perceived health status, having had a coronavirus infection, fear of deterioration of one's health after starting work with coronavirus patients, and fear of contracting the disease while working with coronavirus patients, and using tranquilizers. Level of state anxiety significantly correlated with state anxiety, the self-assessment of professional preparedness for work with coronavirus patients, self-perceived health status, vaccination against coronavirus, and the assistance of a psychiatrist in the past.

**Conclusions:**

The proportion of students showing social anxiety is alarming. Anxiety among the evaluated students during the COVID-19 pandemic was correlated with many factors.

## Introduction

Anxiety disorders are among the most common mental disorders and are associated with a number of short- and long-term impairments and handicaps, including the functioning of personality (1, 2). They are estimated to be the sixth and most important factor leading to non-fatal health loss; moreover, they are among the first ten causes of years lived with disability (YLD) in all the WHO regions (3). Important clinical disorders include e.g., panic disorder, social anxiety disorder, generalized anxiety disorder and specific phobia (4).

Anxiety disorders are considered to be complicated conditions, whose etiology has been only partially understood. Studies indicate that their development is determined by numerous factors, including psychological, genetic, environmental, chemical and biological ones, as well as by the epigenetic relationships between them (5, 6).

Anxiety disorders carry a significant social burden, due to their high prevalence in the adult population, but also in children and adolescents across the world (7, 8). It is an exhausting mental condition characterized by a considerable number of cognitive and somatic symptoms, and it is associated with significant comorbidity and prevalence. Patients suffering from anxiety disorders demonstrate a higher incidence of various medical problems throughout their lives. Moreover, chronic conditions increase the risk of mental disorders and general dysfunction (9–11).

The incidence of anxiety disorders in patients with medical conditions is high, up to 30% in patients with cardiovascular diseases, 29% in patients with epilepsy, 47.0% in people with diabetes, 30.1% in patients with Parkinson's disease, and 48.9% in patients with multiple sclerosis. The most common anxiety disorders in patients with somatic conditions are generalized anxiety disorder and panic disorder (12).

The COVID-19 pandemic has brought into focus the mental health of various affected populations. It is known that the prevalence of epidemics accentuates or creates new stressors, including fear and worry for oneself or loved ones, constraints on physical movement and social activities due to quarantine, and sudden and radical lifestyle changes (13). Anxiety is the most typical manifestation of the acute stress disorder pandemic. It may develop as a result of confrontation with the effects of a pandemic that we cannot cope with: the risk of infecting our close ones, witnessing the death of patients after all the known treatment methods appeared to be ineffective, and the associated sense of helplessness. Anxiety during the coronavirus pandemic may be considered justified, as it is a life-threatening situation.

The outbreak of COVID-19 pandemic adversely affected the mental health of many people, including students. The pandemic increased the level of restlessness, anxiety, and fear for the future in many peoples. Studies indicate that in 71% of subjects, various degrees of anxiety are observed, and 44% also demonstrate symptoms of generalized anxiety (14). Also, Cao et al. (15) assessed anxiety using the GAD-7 scale and found that the risk of infection, including for family members, was a significant contributor to college students' increased anxiety. Although several previous studies have assessed mental health issues during epidemics, most have focused on health workers, patients, children, and the general population (16, 17).

A few studies have been published on the mental well-being of students during a pandemic. However, we still know little about students' mental health during the pandemic. Therefore, both in Poland and globally, there is a considerable information gap in this area. At the same time, data regarding the mental well-being of students appear to be of much importance in the context of certain preventive measures a potential future diseases in this social group.

Considering the above, the main objective of this study was to analyse the level of anxiety in students starting work during a pandemic, with a particular focus on the socio-demographic sources of variance of the psychological indicators. Unfortunately, to our best knowledge, no similar study has been conducted in Poland.

Its results will enable an analysis of the prevalence of anxiety disorders according to subjects' fields of study.

The following hypotheses were proposed:

- The prevalence of anxiety in students starting work during a pandemic is relatively high,
- Vaccinated individuals will demonstrate a lower prevalence of anxiety disorders compared to unvaccinated.
- In order to verify these hypotheses, the following research questions were formulated:
- What is the actual prevalence of anxiety among students starting work during the pandemic?
- How does the prevalence of anxiety among students starting work during the pandemic vary according to gender, age, place of residence, marital status, and graduation in a particular field of study?

## Materials and methods

### Subjects

The study involved 255 (137 females and 118 males) students from Poland starting working with coronavirus patients during the pandemic. The mean age of students was 24.30 ± 1.69. Over half of the subjects (63.1%) lived in a town with a population of over 50 thousand. Over three-quarters of students (78.4%) studied nursing (30.2%), medicine (24.7%), and paramedic science (23.5%). The highest number of respondents (38.4%) were in their 4th year of studies. Detailed socio-demographic characteristics are presented in Table 1.

**Table 1:** Socio-demographic characteristics of respondents.

**Variables**		**participants**
		***N* = 255**
**Age (years), mean SD**		
		24.30 ± 1.69
**Gender**, ***n*** **(%)**		
Female		137 (53.7)
Male		118 (46.3)
**Place of residence**, ***n*** **(%)**		
Rural area		40 (15.7)
A town with a population of up to 50,000		54 (21.2)
Town with a population of 50,000 to 100,000		100 (39.2)
A town with a population of over 100,000		61 (23.9)
**Field of study**, ***n*** **(%)**		
Nursing		77 (30.2)
Medicine		63 (24.7)
Paramedic science		60 (23.5)
Midwifery		30 (11.8)
Physiotherapy		18 (7)
Cosmetology		2 (0.8)
Electroradiology		2 (0.8)
Psychology		2 (0.8)
Social sciences		1 (0.4)
**Year of study**, ***n*** **(%)**		
1	1	0.4
2	1	0.4
3	47	18.4
4	98	38.4
5	64	25.1
6	44	17.3
**Marital status**, ***n*** **(%)**		
Unmarried		115(45.1)
Unmarried partnership		117 (45.9)
Married		23 (9)

### Study design and data collection

The cross-sectional study was conducted between May 1, 2021, and May 30, 2022. Invitations were sent to students starting work during the pandemic and through student portals. Therefore, random sampling was used in this study, i.e., the method of selection where chance determines which subject will be selected from the studied population so that the sample may include any subjects with the same established probability.

The study used the following tools: our original questionnaire (including socio-demographic data, and questions on self-assessment of the anxiety due to working with coronavirus patients, satisfaction due to starting work with coronavirus patients, previous work, self-assessment of one's health status, previous coronavirus infection, vaccination, using tranquilizers, and assistance of a psychologist or psychiatrist in the past (**Tables 3**–**6**), Liebowitz Social Anxiety Scale, and STAI – State-Trait Anxiety Inventory. Using validated scales allowed us to compare our results with those obtained in similar, representative studies conducted in other countries. In addition, correlations between the results obtained according to various scales were analyzed.

The following inclusion criteria were used in the study: student status, starting work at healthcare during a pandemic, and informed consent of a subject for participation in the study. The exclusion criteria included lack of consent for participation in the study, not being a student, and not starting work at a healthcare facility during a pandemic.

Respondents were selected using non-probability random sampling. Concerning the total number of questionnaires returned (308), the rate of full completion was 82.79% (255). The remaining 53 surveys (17.20%) were incomplete.

The link to the survey was placed on numerous social media platforms for students and was sent directly to students starting work during the pandemic. The responses were registered using Google Forms and downloaded as raw data prepared for statistical analysis. Subjects could withdraw from the study at any time.

### Scales

#### Liebowitz social anxiety scale

Liebowitz Social Anxiety Scale (LSAS) is a diagnostic scale frequently used to assess the presence and severity of social phobia symptoms and changes in social anxiety symptoms during therapy (18–20). Genetic and environmental factors, e.g., traumatic experiences, significantly contribute to developing a social phobia. Moreover, catastrophic life experiences play an important role in the etiology of these disorders. In particular, the accumulation of difficult situations has a negative effect. 50% of patients with social phobia can identify the situation that started the disease. According to the conditioning theory, anxiety results from the experience of threats. The scale comprises 24 items that measure the past week's fear and avoidance of social situations. Eleven elements pertain to social interactions, and 13 elements concern public performance. Each item is scored using two 4-point Likert scales. The first score measures anxiety/fear, ranging from 0 (absence) to 3 (severe). The second score measures avoidance, ranging from 0 (never) to 3 (usually 68–100%). The overall score is calculated by adding the total fear and avoidance scores. The Liebowitz Social Anxiety Scale demonstrates good psychometric accuracy, as evidenced by test-retest reliability analysis, internal consistency analysis, convergent validity, and discriminant validity. Respondents were asked to read descriptions of all the situations presented in the table and in relation to each of them, answer two questions: how intense anxiety or fear they experience in this situation and how much they are likely to avoid such a situation, by assigning a score of 0, 1, 2, or 3, based on one's feelings. Next, the results in the “fear or anxiety” column and in the “avoidance” column were summed up, to provide “total scores.” A score of 0–54 points indicated a lack of social phobia; 55–65 indicated a mild social phobia; 66–80 indicated a moderate social phobia; 81–95 indicated a marked social phobia; and 96 or more –severe social phobia.

#### State-trait anxiety inventory (STAI)

Anxiety was assessed using a Polish version of the original Spielberger STAI, typically referred to as STAI-X (21–23). STAI-X is a widely used self-report inventory with two parts, each comprising 20 elements. The State-Trait Anxiety Inventory is designed to measure anxiety in its transitory aspect, “state anxiety” (STI-X1), as well as in the more generalized and persistent “trait anxiety” aspect (STAI-X2) (21, 23, 24). STAI-X1 assesses respondents' emotional state “at the moment,” whereas STAI-X2 shows respondents “how they feel in general.” Each element is scored on a 4-point Likert scale, and respondents can choose from 1 (“not at all”) to 4 (“very much so”) concerning the state subscale, and concerning the trait scale, from 1 (“almost never”) to 4 (“almost always”). The anxiety level is expressed by the number of points obtained by adding the scores in each subscale. The scores for each subscale may range from 20 (low anxiety) to 80 points (high anxiety). A total score of 40 or higher indicates anxiety. A higher score correlates with more significant anxiety. The State-Trait Anxiety Inventory is sensitive to an individual's level of anxiety. It is reliable in patients with specific phobia, panic disorder, social phobia, generalized social phobia, post-traumatic stress disorder, generalized anxiety disorder, obsessive-compulsive disorder, and acute stress disorders (25). In a group of adult females and males, the scale's reliability, measured using the internal consistency coefficient, oscillates between 0.76 and 0.92, while its theoretical validity is 0.51 and 0,57 for males and females, respectively.

### Procedure and ethical issues

The study was conducted after receiving approval by the Bioethical Committee of the Medical University of Bialystok, Poland (no. APK.002.330.2021).

### Statistical analysis

Data were processed using Microsoft Excel 2013 spreadsheet and analyzed using Statistica PL version 13.0. The descriptive statistics were used to describe participants' demographics (age, gender, academic year, and place of residence). The Shapiro-Wilk test evaluated the data for normal distribution since the data (the anxiety questionnaires) did not show normal distribution. In addition, the non-parametric Spearman's Rank Correlation Test was used. The significance level was taken as *p* < 0.05 in the study.

## Results

In the Liebowitz Social Anxiety Scale, respondents received a mean score of 52.9 ± 31.3 points, which generally indicates a lack of social phobia; however, 51% of subjects demonstrated mild to severe social phobia symptoms. In addition, females had significantly (*p* < 0.05) higher scores of anxiety on the Liebowitz Social Anxiety Scale compared to males (Table 2).

**Table 2:** Levels of social anxiety, state anxiety (X-1), and trait anxiety (X-2) according to gender, age, place of residence, field of study, year of study, and marital status.

	**Liebowitz social anxiety**			**State-trait anxiety inventory**					
	**scale**								
				**STAI X-1**			**STAI X-2**		
	**Mean**	**Min**.	**Max**.	**Mean**	**Min**.	**Max**.	**Mean**	**Min**.	**Max**.
**Gender**									
Female	56.4 ± 29.9	0	144	50.0 ± 1.9	44	56	49.4 ± 2.8	41	59
Male	48.7 ± 32.7	0	126	50.0 ± 1.9	46	56	48.7 ± 2.6	42	56
ρ-value	NS			NS			0.134		
*P*-value							<0.05		
**Age**									
	48.9 ± 26.3	2	144	50.2 ± 1.9	47	53	49.7± 3.14	43	55
ρ-value	0.124			NS			0.287		
*P*-value	0.04						<0.001		
**Place of residence**									
Town with a population of up to 50,000	51.6 ± 35	3	144	5.00 ± 2.0	44	54	49.3 ± 3.0	43	59
Town with a population of 50,000 to 100,000	54.2 ± 29.1	0	106	50.0 ± 1.9	44	55	49 ±2.5	43	58
Town with a population of over 100,000	58.2 ± 29.9	2	126	50.0 ± 2.0	45	56	49.1 ± 2.8	42	56
Village	43.0 ± 33.4	0	97	50.3 ± 1.9	46	56	49.0 ± 2.9	41	55
ρ-value *P*-value	NS			NS			NS		
**Field of study**									
Electroradiology	67.5 ± 30.4	46	89	49.5 ± 0.7	49	50	47.5 ± 7.8	42	53
Physiotherapy	44.4 ± 34.5	0	99	50.1 ± 1.6	46	52	48.2 ± 1.9	44	53
Cosmetology	82 ± 25.5	64	100	52 ± 0	52	52	51.5 ± 4.9	48	55
Medicine	46.1 ± 29.9	0	108	49.6 ± 1.6	45	54	49.7 ± 2.3	45	59
Nursing	58.5 ± 31.4	0	108	50.0 ± 2.0	44	55	48.7 ± 3.1	41	58
Obstetrics	59.8 ± 31.9	0	144	50.0 ± 1.7	45	53	48.9 ± 2.9	42	55
Paramedic science	50.0 ± 31.3	0	126	50.2 ± 2.2	44	56	49.1 ± 2.5	43	56
Social sciences	86.0 ± 0	86	86	56.0 ± 0	56	56	52.0 ± 0	52	52
Psychology	44.5 ± 21.9	29	60	52.0 ± 1.4	51	53	51.0 ± 0	51	51
ρ-value *P*-value	NS			NS			NS		
**Year of study**									
1 year	22.0 ± 0	22	22	52.0 ± 0	52	52	47.0 ± 0	0	0
2 year	126.0 ± 0	126	126	54.0 ± 0	54	54	52.0 ± 0	0	0
3 year	36.6 ± 29.6	0	104	51.2 ± 1.7	49	56	49.8 ± 3.0	41	59
4 year	56.1 ± 33.8	0	144	50.0 ± 1.9	44	55	48.9 ± 2.7	43	58
5 year	56.0 ± 28.4	0	105	49.7 ± 2.0	44	56	48.5 ± 2.9	42	56
6 year	57.4 ± 26.1	0	108	49.2 ± 1.5	45	53	49.6 ± 1.7	45	53
ρ-value *P*-value	NS			NS			NS		
**Marital status**									
Unmarried	48.0 ± 31.2	0	108	50.1 ± 2.1	44	56	49.5 ± 2.5	41	59
Married	56.4 ± 34.2	0	108	50.0 ± 2.5	45	53	48.0 ± 2.5	44	51
Unmarried partner	56.9 ± 30.8	0	144	49.9 ± 1.6	44	55	48.9 ± 2.8	42	58
ρ-value	0.123			NS			NS		
*P*-value	<0.05								

Spearman's Rank Correlation Test, ρ = Spearman's rank correlation coefficient, NS- not significant.

Level of trait anxiety significantly correlated with age and gender (females). The level of social was associated significantly with marital status. The results are illustrated in Table 2.

We also found a significant correlation between the self-assessment of the experienced fear on a 5-point scale and social anxiety. Details are shown in Table 3.

**Table 3:** Level of social anxiety and intensity of state anxiety (X-1) and trait anxiety (X-2) according to self-perceived anxiety due to starting work with coronavirus patients.

	**Liebowitz social anxiety**			**State-trait anxiety inventory**					
	**scale**								
				**STAI X-1**			**STAI X-2**		
	**Mean**	**Min**.	**Max**.	**Mean**	**Min**.	**Max**.	**Mean**	**Min**.	**Max**.
**Self-assessment of the anxiety due to working with coronavirus patients**									
Yes	61.9 ± 27.3	9	105	50.0 ± 2.4	46	55	49.5 ± 3.3	47	59
Sometimes	59.9 ± 28.0	0	144	49.9 ± 1.9	44	56	48.9 ± 2.7	41	56
No	36.8 ± 33.7	0	126	50.3 ± 1.8	45	55	49.3 ± 2.3	42	55
I do not know	19.3 ± 12.4	6	36	50.0 ± 0	50	50	46.3 ± 3.3	43	50
ρ-value *P*-value	NS			<0.001			NS		
**Self-assessment of the experienced fear on a 5-point scale**									
0	33.2 ± 31.4	0	98	50.5 ± 1.7	46	55	49.2 ± 2.3	44	55
1	52.9 ± 37.5	0	144	50.1 ± 2.0	44	54	48.8 ± 2.9	41	55
2	56.5 ± 27.0	0	105	49.7 ± 1.7	46	56	49.0 ± 2.7	42	56
3	62.0 ± 23.4	9	108	50.0 ± 2.3	44	56	49.4 ± 2.0	45	55
4	80.4 ± 17.5	61	97	48.6 ± 1.0	48	50	49.4 ± 5.0	43	58
5	82.5 ± 23.3	66	99	53.5 ± 0.7	53	54	51.5 ± 10.6	44	59
ρ-value	0.337			NS			NS		
*P*-value	<0.001								

Spearman's Rank Correlation Test, ρ = Spearman's rank correlation coefficient, NS- not significant.

We found a significant correlation between the self-assessment of professional preparedness for work with coronavirus patients and state anxiety. The results are shown in Table 4.

**Table 4:** Level of social anxiety, state anxiety (X-1) or trait anxiety (X-2) and satisfaction with starting work with coronavirus patients, previous work experience in healthcare facilities, self-perceived professional preparedness for work with coronavirus patients and motivation for starting such work.

	**Liebowitz social anxiety**			**State-trait anxiety inventory**					
	**scale**								
				**STAI X-1**			**STAI X-2**		
	**Mean**	**Min**.	**Max**.	**Mean**	**Min**.	**Max**.	**Mean**	**Min**.	**Max**.
**Satisfaction due to starting work with coronavirus patients**									
Yes	53.4 ± 31.3	0	144	49.9 ± 1.8	44	56	49.0 ± 2.6	41	58
No	49.9 ± 33.6	4	86	49.6 ± 3.6	43	52	48.6 ± 3.0	43	52
I do not know	45.9 ± 35.1	0	126	51.5 ± 1.8	48	54	50.6 ± 3.8	45	59
ρ-value *P*-value	NS			NS			NS		
**Previous work in healthcare facilities**									
No	54.2 ± 32.3	0	144	49.9 ± 2.0	44	56	49.3 ± 2.7	41	59
Yes	50.2 ± 28.0	0	105	50.3 ± 1.6	46	55	48.5 ± 2.7	43	55
I do not remember	5.0 ± 1.4	4	6	51.0 ± 1.4	50	52	47.0 ± 5.7	43	51
ρ-value *P*-value	NS			NS			NS		
**Self-assessment of professional preparedness for the work with coronavirus patients**									
No	48.2 ± 31.5	0	144	50.7 ± 2.1	45	56	49.4 ± 2.4	44	55
Yes	53.4 ± 32.3	0	108	46.7 ± 1.9	44	55	49.1 ± 2.7	41	59
I do not know	55.7 ± 30.0	0	126	50.0 ± 1.6	46	56	48.8 ± 2.9	42	56
ρ-value *P*-value	NS			<0.001			NS		
**Factors affecting the decision to work with coronavirus patients**									
Persuaded by colleagues	65.8 ± 30.3	0	108	49.3 ± 1.9	44	53	48.7 ± 3.2	43	56
Own decision	60.2 ± 29.5	0	144	49.7 ± 1.9	44	56	49.1 ± 2.7	41	58
Hard to say	35.7 ± 28.3	0	126	50.8 ± 1.8	46	56	49.2 ± 2.5	42	58
ρ-value *P*-value	NS			NS			NS		

Spearman's Rank Correlation Test, ρ = Spearman's rank correlation coefficient, NS- not significant.

Statistically significant relationships were found between the level of social anxiety or state anxiety and self-perceived health status, having had a coronavirus infection, fear of deterioration of one's health after starting work with coronavirus patients, and fear of contracting the disease while working with coronavirus patients, as well as between state anxiety and self-perceived health status or state anxiety and having been vaccinated against coronavirus. The results are presented in Table 5.

**Table 5:** Level of social anxiety, state anxiety (X-1) or trait anxiety (X-2) and self-perceived health status, fear of health deterioration after starting work with coronavirus patients, having had a coronavirus infection, having received coronavirus vaccination and fear of contracting the disease while working with coronavirus patients.

	**Liebowitz social anxiety**			**State-trait anxiety inventory**					
	**scale**								
				**STAI X-1**			**STAI X-2**		
	**Mean**	**Min**.	**Max**.	**Mean**	**Min**.	**Max**.	**Mean**	**Min**.	**Max**.
**Self-assessment of one's health status**									
Very good	58.8 ± 33.2	0	105	49.9 ± 2.2	44	55	48.9 ± 2.9	42	56
Well	51.8 ± 31.7	0	144	50.0 ± 1.9	44	56	49.3 ± 2.5	41	59
Moderate	50.2 ± 27.9	5	126	50.2 ± 1.7	46	55	48.9 ± 2.9	43	55
Poor	41.8 ± 30.9	4	68	49.5 ± 2.6	46	52	50.5 ± 0.6	50	51
I do not know	6.0 ± 0	6	6	50.0 ± 0	50	50	43.0 ± 0	43	43
ρ-value	0.132			NS			NS		
*P*-value	<0.05								
**Fear of deterioration of the health status after starting work with coronavirus patients**									
Yes	63.2 ± 26.3	0	108	49.6 ± 2.0	44	55	49.0 ± 3.0	41	59
No	43.5 ± 34.2	0	144	50.4 ± 1.8	45	56	49.3 ± 2.5	42	56
I do not know	50.9 ± 27.0	0	97	50.3 ± 1.9	47	56	48.8 ± 2.6	43	54
ρ-value	0.251			0.185			NS		
*P*-value	<0.001			<0.01					
**Previous coronavirus infection**									
Yes	59.3 ± 29.2	0	108	49.8 ± 2.0	44	56	48.8 ± 2.8	41	48
No	46.9 ± 32.8	0	144	50.1 ± 1.9	45	55	49.6 ± 2.5	43	59
I do not remember	44.7 ± 32.1	0	106	50.5 ± 1.5	48	56	48.9 ± 2.8	42	55
ρ-value	0.219			0.130			NS		
*P*-value	<0.001			<0.05					
**Vaccination against coronavirus**									
No	41.1 ± 35.7	0	126	50.0 ± 2.0	46	54	49.5 ± 3.1	43	55
Two doses	52.2 ± 31.1	0	144	50.0 ± 2.0	44	56	49.2 ± 2.6	42	59
One dose	56.2 ± 29.0	3	108	50.1 ± 1.5	46	54	48.3 ± 3.0	41	56
Single-dose vaccine	58.9 ± 32.7	0	108	49.9 ± 2.0	44	55	49.3 ± 2.7	43	55
ρ-value *P*-value	NS			NS			NS		
**Fear of contracting the disease while working with coronavirus patients**									
I am not afraid	31.6 ± 29.5	0	126	50.6 ± 2.0	45	56	49.5 ± 2.1	44	55
Yes, to a lesser degree than with other diseases	50.0 ± 19.8	36	64	51.0 ± 1.4	50	52	52.5 ± 3.5	50	55
Yes, to the same degree as with other diseases	57.1 ± 30.6	0	144	50.0 ± 1.6	44	56	48.9 ± 2.7	41	56
Yes, to a higher degree than with other diseases	65.6 ± 25.4	9	108	49.5 ± 2.2	44	45	49.1 ± 3.0	43	59
I do not know	34.4 ± 28.9	0	60	50.2 ± 2.5	43	52	48.2 ± 3.3	43	52
ρ-value *P*-value	NS			NS			NS		

Spearman's Rank Correlation Test, ρ = Spearman's rank correlation coefficient, NS- not significant.

Also, significant correlations were detected between the level of social anxiety or state anxiety and using tranquilizers and the assistance of a psychiatrist in the past. The results are presented in Table 6.

**Table 6:** Level of social anxiety, state anxiety (X-1) or trait anxiety (X-2) and use of tranquilizers or receiving assistance of a psychologist and/or psychiatrist in the past.

	**Liebowitz social anxiety**			**State-trait anxiety inventory**					
	**scale**								
				**STAI X-1**			**STAI X-2**		
	**Mean**	**Min**.	**Max**.	**Mean**	**Min**.	**Max**.	**Mean**	**Min**.	**Max**.
**Using tranquilizers**									
No	47.0 ± 29.4	0	126	50.2 ± 1.8	45	56	49.0 ± 2.5	42	59
Yes	68.3 ± 27.0	0	108	49.3 ± 2.1	44	56	49.3 ± 2.9	41	58
I do not know	55.1 ± 46.5	0	144	50.6 ± 1.3	49	55	49.2 ± 3.6	43	55
ρ-value	0.287			0.194			NS		
*P*-value	<0.001			<0.01					
**Assistance of a psychologist in the past**									
No	52.4 ± 31.9	0	144	50.0 ± 2.0	44	56	49.2 ± 2.6	41	59
Yes	55.5 ± 30.0	0	126	50.1 ± 1.7	45	54	49.0 ± 2.8	42	58
I do not remember	5.0 ± 1.4	4	6	51.0 ± 1.4	50	52	47.0 ± 5.7	43	51
ρ-value *P*-value	NS			NS			NS		
**Assistance of a psychiatrist in the past**									
No	53.2 ± 31.6	0	144	50.0 ± 1.9	44	56	49.1 ± 2.7	41	59
Yes	57.8 ± 7.4	47	64	52.0 ± 0.8	50	55	51.5 ± 2.4	50	55
I do not remember	5.0 ± 1.4	4	6	51.0 ± 1.4	50	52	47.0 ± 5.6	43	51
ρ-value	NS			0.163			NS		
*P*-value				<0.01					

Spearman's Rank Correlation Test, ρ = Spearman's rank correlation coefficient, NS- not significant.

## Discussion

In our study, aimed at verifying whether students who start work during a pandemic risk developing anxiety, 51% of participating students demonstrated symptoms of mild to severe social phobia. Students' level of anxiety correlated significantly with age, gender (i.e., woman), marital status, self-assessment of fear experienced, health status, having been infected with COVID-19, fear of deteriorating personal health after beginning to work with patients with COVID-19, fear of contracting COVID-19 while working with such patients, and the use of tranquilizers.

During an epidemic or pandemic, symptoms of specific phobias may develop or worsen. Specific phobias, thought to be common disorders, may affect approximately 20% of the population (24). In every phobia, anticipatory anxiety over and the avoidance of the feared situation can occur before or during an anxiety-inducing event.

In our study, 43.5% of students feared the deterioration of their health status after beginning to work with patients with COVID-19. That result aligns with past findings (25, 26), including that, in China, more than half of a similar sample had symptoms of depression, while 44.6% had symptoms of anxiety, 34% of insomnia, and 71.5% of distress (27).

In our study, the mean State–Trait Anxiety Inventory (STAI) score for state anxiety was 50.0 ± 1.9 points, which indicates a high level of subjective anxiety and tension, along with a predisposition to anxious reactions. That result is consistent with the findings of García-González et al. (28), who had university students complete the STAI online in the first and fourth weeks of their study during the COVID-19 pandemic. Total anxiety levels had increased by the fourth week compared with the 1st week (first week: 50.4 ± 20.8; fourth week: 59.9± 10.6). In addition, the authors' linear regression model demonstrated that the significant predictors for state anxiety were being a woman and current year of study.

According to literature analyzed in a systematic review by, among others, scientists from the Norwegian Institute of Public Health during the COVID-19 pandemic, up to 97% of healthcare workers have experienced distress or stress that adversely affected their readiness to act and their mental health (29).

In other research, Lasheras et al. (30) investigated the prevalence of anxiety among medical students in China during the COVID-19 pandemic. In their systematic review and meta-analysis, only eight studies were ultimately included for qualitative and quantitative analysis, which revealed a prevalence of anxiety of 28%, with significant heterogeneity between the studies. Although that reported prevalence of anxiety among medical students is similar to the prevalence before the pandemic, it correlates with several specific COVID-related stressors. Furthermore, the authors found that students comprised 89% of the total sample of the meta-analysis, which could have compromised the external validity of their work.

Another systematic review and meta-analysis (31) evaluating the prevalence of mental health problems and sleep disturbances among nursing students during the COVID-19 pandemic included 17 studies representing 13,247 nursing students. The prevalence of four health problems and sleep disturbances was identified, the most prevalent being depression (52%). Other COVID-19-related health problems were fear (41%), anxiety (32%), stress (30%), and sleep disturbances (27%).

In another systematic review, Santabarbara et al. (32) assessed the prevalence of anxiety among dental students during the outbreak of COVID-19. In their sample of 15 studies, anxiety had been reported by 35% of dental students, independent of gender, response rate, and methodological quality. Furthermore, they detected a lower prevalence of anxiety in studies conducted in Europe than in ones conducted on other continents.

Another systematic review (33) including 70 studies representing 101,017 participants evaluated healthcare workers' anxiety, depression, trauma, and sleep disorders during the COVID-19 pandemic. The results showed that the estimated pooled rates of prevalence were 30.0% for anxiety, 31.1% for depression, 56.5% for acute stress, 20.2% for post-traumatic stress disorder, and 44.0% for sleep disorders. Three factors—proportion of females, proportion of nurses, and location—were found to be sources of heterogeneity in the sub-group and meta-regression analysis.

Our results suggest that although it is important for everyone to learn how to cope with sudden exposure to stress and anxiety (34), it is especially crucial for young people who are beginning their professional careers. Individuals continuously exposed to stress more frequently experience increased mental tension, which reduces their mental comfort and adversely affects their social and family relations. Therefore, it is essential to ensure that young, entry-level healthcare workers can consult a specialist who can help them to develop their methods of coping with stress and/or anxiety and with the burden associated with working in healthcare.

The novelty of the study is that we evaluated the level of anxiety among students who start work during the COVID-19 pandemic, not only in relation to the socio-demographic factors but to many other factors (e.g., health status, vaccination, coronavirus infection, assistance of a psychologist or psychiatrist in the past, and using tranquilizers).

## Conclusions

1. The proportion of students exhibiting social anxiety is alarming.
2. Anxiety during the COVID-19 pandemic among the students evaluated was correlated with many factors.
3. Significant correlations were demonstrated between anxiety and being a woman, age, marital status, self-perceived anxiety due to beginning to work with patients with COVID-19, self-perceived professional preparedness for working with such patients, self-perceived health status, having been infected with COVID-19, fear of deteriorating health after beginning to work with patients with COVID-19, fear of contracting COVID-19 while working with such patients, having been vaccinated against COVID-19, and the use of tranquilizers.

### Study limitations

This study has several limitations. First, it was a cross-sectional study based on online survey questionnaires. Second, the study group was too small to generalize the outcomes to the entire population of students starting work during the COVID-19 pandemic. Third, nursing, medicine and paramedic students were overrepresented in the study group, so the results should be verified in an equally numerous group of students representing other fields of study. However, most students specializing in these fields could have direct contact with COVID-19 patients.

Nevertheless, this study focused on a general assessment of anxiety disorders among students starting work during a pandemic. As a research team, we will make efforts to study groups with a more balanced representation of students from individual medical fields in our future research protocols. Despite these limitations, the outcomes of this study may provide a starting point for further studies on the prevalence of anxiety disorders among students starting work during a pandemic and the socio-demographic determinants of such disorders. Furthermore, this study confirms the need for such studies, as – on average – one in two students would ask for psychological or psychiatric assistance, if necessary.

## Data availability statement

The original contributions presented in the study are included in the article/supplementary material, further inquiries can be directed to the corresponding author/s.

## Ethics statement

The studies involving human participants were reviewed and approved by the BioEthical Committee of the Medical University of Bialystok (resolution no. APK.002.330.2021). The patients/participants provided their written informed consent to participate in this study.

## Author contributions

Conceptualization, formal analysis, methodology, writing—original project, and writing—review and editing: KC, EK-K, and WK. Data curation, acquisition of financing, and project administration: EK-K. Investigation: KC and EK-K. All authors contributed to this article and approved the submitted version.

## Conflict of interest

The authors declare that the research was conducted in the absence of any commercial or financial relationships that could be construed as a potential conflict of interest.

## Publisher's note

All claims expressed in this article are solely those of the authors and do not necessarily represent those of their affiliated organizations, or those of the publisher, the editors and the reviewers. Any product that may be evaluated in this article, or claim that may be made by its manufacturer, is not guaranteed or endorsed by the publisher.
